# Vitamin a modulates neurogenesis-associated pathways and cholinergic signaling in Alzheimer’s disease: potential role of reactive astrocytes via NGN2/SOX-11 and SIRT-1

**DOI:** 10.1038/s41598-026-55386-z

**Published:** 2026-07-25

**Authors:** Nesrine Saeid El-Mezayen, Yara Alaa Eesa, Wed Alaa Hassan, Dina Ahmed Aly, Yassmen Soliman Saafan, Eman Mahmod Khedr, Aliaa Sherief, Eman Ali Elkordy

**Affiliations:** 1 Department of Pharmacology, Faculty of Pharmacy, Pharos University in Alexandria, Alexandria, Egypt; 2 Faculty of Pharmacy, Pharos University in Alexandria, Alexandria, Egypt; 3 Department of Anatomy and Physiology, College of Medicine, Imam Mohammad Ibn Saud Islamic University (IMSIU), Riyadh, Saudi Arabia

**Keywords:** Alzheimer’s disease, Astrocytes, Neurogenesis, NGN2, SIRT-1, SOX-11, Vitamin A, Diseases, Drug discovery, Neurology, Neuroscience

## Abstract

Alzheimer’s disease (AD) is a progressive neurodegenerative disorder lacking effective disease-modifying therapies. A promising regenerative approach involves enhancing endogenous neurogenic capacity within the injured brain. Reactive astrocytes—stellate-like cells in the AD brain—may contribute to a pro-neurogenic environment through transcription factors (TFs) such as neurogenin 2 (NGN2) and SOX-11. This process is tightly regulated by epigenetic mechanisms, particularly SIRT-1, a neuroprotective histone deacetylase that modulates TF activity and neuronal fate. Vitamin A (V_A_), a key regulator of differentiation and epigenetic remodeling via its active metabolite retinoic acid, is stored in astrocytes and hepatic stellate cells (HSCs). We hypothesized that AD-related astrocyte activation depletes cerebral V_A_, mobilizes hepatic stores, contributes to liver fibrosis, and that V_A_ supplementation may restore astrocytic function, activate endogenous TFs via SIRT-1, and drive cholinergic neuron regeneration. In a scopolamine (SCO)-induced AD rat model, V_A_ biodistribution was traced using confocal microscopy. Brain and liver V_A_ deficiency were confirmed via retinol-binding protein (RBP) and ALDH1A1 expressions. Rats received V_A_ (1500, 3000, or 4500 IU/kg/day) or donepezil. Outcomes included neurogenesis (DCX), NGN2/SOX-11 expression, SIRT-1 activation, cholinergic regeneration, amyloid-β deposition, and serum tau. Liver fibrosis was assessed via TGF-β, hydroxyproline and histopathologically. AD induced systemic V_A_ depletion and liver fibrosis. Medium-dose V_A_ (VAMD) significantly enhanced neurogenesis, TF expression, SIRT-1 activation, cholinergic regeneration, and reversed liver fibrosis. VAMD demonstrated neuroregenerative and antifibrotic effects, indicating a possible therapeutic role in AD.

## Introduction

Alzheimer’s disease (AD) is the most common form of neurodegenerative dementia, characterized by progressive cognitive decline and neuronal loss. Despite advances in neuroimaging, biomarker development, and multi-omics technologies, the exact molecular mechanisms underlying AD pathogenesis remain elusive, and no single hypothesis can fully account for the complexity of the disease^1^. While the pathological hallmarks of AD, extracellular amyloid-β (Aβ) plaques and intracellular neurofibrillary tangles composed of hyperphosphorylated tau, have dominated research for decades^2^^,^ these lesions alone do not fully explain the extent of clinical symptoms or disease heterogeneity. Recent perspectives have shifted toward a broader understanding of AD as a disorder of neural circuits, implicating mitochondrial dysfunction, oxidative stress, impaired proteostasis, neurovascular impairment, and neuroinflammation as significant contributors to its pathogenesis^3^. Within this complex network failure, multiple neuronal subtypes are affected, including glutamatergic, GABAergic, and monoaminergic systems. However, among these, cholinergic neurons are disproportionately vulnerable and are among the earliest to degenerate in AD^3^. Their loss correlates strongly with impairments in memory, attention, and executive function, and underlies the rationale for current symptomatic treatments targeting cholinergic transmission, either increasing synaptic acetylcholine (ACh), such as acetylcholine esterase inhibitors (AChEIs), as donepezil (DON), or acting as N-methyl-D-aspartate (NMDA) antagonists (e.g., memantine)^4^. Given their early and central role in cognitive processing and disease progression, this study focuses specifically on targeting the cholinergic system.

A true disease-modifying therapy for AD would require the restoration of lost neuronal populations, particularly cholinergic neurons. While embryonic stem cells (ESCs) and induced pluripotent stem cells (iPSCs) have been investigated as sources for neuron replacement in both preclinical and early clinical settings^5–8^^,^ their application remains limited by serious concerns, including ethical issues, risk of tumorigenesis, immune rejection, and genomic instability^9^. In contrast, direct reprogramming of somatic cells, such as fibroblasts or astrocytes, into functional neurons using defined transcription factors (TFs) offers a promising and innovative alternative. This approach bypasses the pluripotent state entirely, thereby minimizing associated risks while enabling in situ neuronal regeneration. By targeting endogenous cell populations and avoiding transplantation, direct reprogramming represents a novel and potentially safer strategy for neuronal restoration in neurodegenerative diseases. This study builds on this emerging paradigm, focusing specifically on the reprogramming of somatic cells into cholinergic neurons as a regenerative approach for AD.

Astrocytes constitute an ample abundance in the mammalian brain. Ascertaining the vital role of astrocytes in synapse formation, maturation, and efficacy, besides their interaction with and sculpturing developing neuronal circuits, puts these cells at a glance^10^. Growing substantiation in recent years indicated the potential of astrocytes to be starter cells for reprogramming into functional neurons, capable of replacing degenerated neurons in AD. In neurodegenerative conditions such as AD, astrocytes become reactive and exhibit enhanced plasticity, which may increase their responsiveness to pro-neurogenic signals^9^. This strategy bypasses many of the major limitations associated with pluripotency induction, including tumorigenic risk, ethical concerns, and immune incompatibility, offering a safer and more efficient route for in situ neuronal regeneration. Nonetheless, the government of exogenous TFs is poor and requires complex time-consuming manoeuvers with low induction efficiency^11^. Accordingly, modulation of endogenous TFs associated with neuronal differentiation may represent a promising alternative approach to support regenerative processes in AD.

Neurogenin 2 (NGN2) and SOX-11 are TFs that play pivotal roles in neuronal identity and fate determination. Notably, both have been shown to successfully reprogram fibroblasts into functional cholinergic neurons, bypassing the proliferative progenitor state and accelerating direct neuronal conversion^12^. However, the expression of these TFs is under tight epigenetic control. For instance, histone deacetylase (HDAC) activity suppresses NGN2 expression in late-stage neural precursor cells, and inhibition of this repression extends the neurogenic phase and delays astrocytic differentiation^10^. Similarly, the aberrant, de novo expression of SOX-11 has been associated with histone modifications, underscoring the role of epigenetic regulation in determining neural fate^13^. Sirtuin 1 (SIRT-1), a class III HDAC, has emerged as a key regulator in neurodegeneration. SIRT-1 exerts neuroprotective effects against Aβ toxicity, modulates multiple transcriptional pathways in the brain, and its deficiency in microglia has been implicated in cognitive decline in AD^14^. These findings suggest that modulating the epigenetic environment can influence TF expression patterns and neuronal outcomes, supporting the potential of targeting TF-associated pathways in regenerative strategies.

Vitamin A (V_A_) and its active metabolite retinoic acid (RA) have been shown to impact both epigenetic and transcriptional regulation in the nervous system. RA directly modulates neural-specific gene expression through nuclear retinoid receptors and promotes the expression of several TFs associated with neuronal differentiation pathways, including those implicated in dopaminergic neuron generation^15,16^. V_A_ also facilitates the derivation of induced pluripotent stem cells (iPSCs)^17^ and enhances neurogenesis through antioxidant, anti-apoptotic, and anti-inflammatory actions, involving pathways such as Nrf2 and glutathione regulation^18^. Despite its wide-ranging neuroprotective properties and reported effects in slowing dementia progression^19^, the potential of V_A_ to modulate neurogenesis-related pathways and support cholinergic regenerative responses in AD remains insufficiently explored.

V_A_ has a distinctive diffuse storage system known as the stellate cell system, present in the liver and extrahepatic organs, including the pancreas, lung, kidney, colon, and heart^20^. Under physiological conditions, hepatic stellate cells (HSCs) store nearly 80% of total body V_A_^21^. Upon tissue injury, HSCs lose their V_A_ reserves and convert into matrix-producing myofibroblast-like cells^22^. This behavior is the foundation for HSC-targeted drug delivery, wherein V_A_-coupled liposomes selectively accumulate in V_A_-deficient activated HSCs to deliver antifibrotic agents^23,24^.

Emerging evidence suggests that astrocytes may share key structural and functional features with HSCs, supporting the idea that they belong to a broader stellate cell-like system. In AD and other neurodegenerative conditions, astrocytes become reactive and adopt a fibrotic, matrix-producing phenotype similar to that of activated HSCs^25^. Moreover, astrocytes are a known source of RA in the brain, regulating key aspects of adult neurogenesis^26,27^. Despite these findings, the co-occurrence of V_A_ deficiency and AD-related astrocyte activation has not yet been explored, nor is it known whether V_A_ can preferentially associate with reactive astrocytes in AD. Demonstrating such targeting could support the development of astrocyte-associated therapeutic strategies using V_A_-coupled approaches. Supporting this hypothesis, previous work showed that Parkinson’s disease (PD) is associated with V_A_ depletion in both liver and brain tissues and that systemic V_A_ administration selectively targeted brain astrocytes in PD models^15^.

This study aimed to evaluate the biodistribution, neurogenesis-associated and cholinergic regenerative effects of V_A_ in a scopolamine (SCO)-induced rat model of AD. V_A_ was administered orally and intraperitoneally, and its distribution was tracked by quantifying intrinsic fluorescence in various organs using confocal microscopy. Cellular uptake in α-SMA–positive reactive glial cells/astrocyte-like cells was assessed by fluorescent labeling. To assess V_A_’s role in neurogenesis-associated signaling, the expression of DCX, SIRT-1 localization, and the gene expression of SOX-11 and NGN2, two TFs associated with neuronal differentiation and cholinergic lineage-related pathways was examined. Functional and structural outcomes were evaluated using histopathological analysis, along with markers of cholinergic transmission and AD pathology. Additionally, we investigated the relationship between V_A_ depletion, AD progression, and liver fibrosis by measuring hepatic and brain V_A_ content, RBP and ALDH1A1 expression, liver histology, and TGF-β and hydroxyproline levels.

## Materials and methods

### Materials

V_A_ (all-trans-retinol), choline/acetylcholine quantification kit and Ach activity assay kit were from Sigma-Aldrich (St. Louis, United States); Donepezil hydrochloride was from Pfizer (New York, USA); Hyoscine (SCO hydrobromide) was obtained from Boehringer Ingelheim, Germany; Rat transforming growth factor-beta (TGF-ꞵ) and ALDH1A1 ELISA Kits from CUSABIO (Houston, United States); Rat choline acetyltransferase ELISA Kit was from Elabscience (Texas, United States); Hydroxyproline ELISA Kit from Mybiosource (California, United States); Tau ELISA kit from Novus Biologicals (Centennial, United States); DCX ELISA kit was from Assay Genie (Dublin, Ireland). Trizole plus RNA purification kit and alfa-smooth muscle actin (α-SMA) antibody were from Invitrogen—ThermoFisher (Carlsbad, United States); Rotor-Gene SYBR® green PCR kit and miScript SYBR® green, miRNeasy Mini total and miRNA extraction kit, high capacity cDNA reverse transcription kit and RT-PCR reagents kits and universal primers were from Qiagen (Hilden, Germany); SIRT-1 antibody (Catalog # sc-74465) was purchased from Santa Cruz Biotechnology, Inc (Texas, United States); Goat anti-mouse IgG H&L (Alexa Fluor® 555) secondary antibody was from Abcam (Cambridge, UK).

### Animals

This study utilized 48 female Sprague–Dawley rats from a locally bred strain, each aged 10–12 weeks and weighing approximately 200 ± 10 g. The animals were obtained from the animal facility of the Faculty of Pharmacy, Pharos University in Alexandria (PUA), Egypt, where they were also maintained throughout the experimental period. All animal handling and experimental protocols were reviewed and approved by the Ethics Committee of Pharos University in Alexandria, and the procedures were conducted in accordance with NIH guidelines for the care and use of laboratory animals.

### The experimental design

#### Assessment of V_A_ bio-distribution in rats with induced AD following oral and intraperitoneal administration

Based on the intrinsic fluorescence of retinol, V_A_ can be fluorescently detected and quantified in different tissues following either oral or systemic administration using confocal microscopy (Leica DMi8, Wetzlar, Germany) in rats with induced AD. This approach allows the assessment of V_A_ distribution across tissues and its relative accumulation in the brain following AD induction, as well as comparison of its distribution patterns after oral versus systemic administration.

AD was induced in 12 rats by chronic daily administration of 1mg kg^-1^ SCO intraperitoneally (i.p) for four weeks^4^. These rats were compared to another 12 normal rats with no AD induction. Twelve rats (six normal and six with induced AD) received four successive oral doses of V_A_ via oral gavage syringe, whereas the other 12 rats (six normal and six with induced AD) received four successive systemic doses of V_A_ i.p. The dosing interval was two hours, and the V_A_ dose was 1500 IU/Kg. Thirty minutes after the final treatment, rats were deeply anesthetized with desflurane inhalation anesthesia (8% in oxygen) delivered via a precision vaporizer in an induction chamber. Adequate depth of anesthesia was confirmed by the absence of the pedal withdrawal reflex. Animals were then euthanized by cervical dislocation under deep anesthesia, followed by rapid dissection and harvesting of the brain, lung, kidney, liver, spleen, and small intestine for subsequent analyses. Harvested organs were cryosectioned, mounted on slides, and fixed in acetone after air drying. Then, slides were examined under confocal microscopy. Nuclei were stained with the nuclear stain, Hoechst, for one minute. The fluorescent images of V_A_ and the nuclei were acquired at emission wavelengths of 695 and 461, respectively. The intensity of V_A_ fluorescence in different sections was semi-quantified using ImageJ software.

#### Assessment of VA uptake in reactive astrocytes

Reactive astrocytes in the cryosectioned brain samples, prepared in the previous section, were stained using a properly diluted α-SMA primary antibody, which was employed as a marker of fibrosis-associated reactive astrocytic activation. Briefly, tissue sections were blocked, incubated with the primary antibody, and then washed with PBS. For fluorescence imaging, sections were incubated with diluted goat anti-mouse IgG H&L (Alexa Fluor® 555) secondary antibody for 30 min at room temperature in the dark. Confocal microscopy examination was performed at emission wavelengths of 550 to detect α-SMA immunofluorescence and at 695 to detect V_A_ fluorescence. Co-localization in merged images was used to evaluate the uptake of V_A_ in α-SMA–positive reactive astrocytes. Images were obtained from comparable anatomical regions of the hippocampus under identical imaging conditions.

#### Experimental grouping

AD was induced using daily 1mg kg^-1^ SCO i.p injections for four weeks^4^ in thirty rats. These rats were divided into five equal groups,each six rats: The positive control group, where rats received 1 mL/day corn oil orally, the donepezil (DON)–treated group, rats received 2.5 mg kg^-1^ day^-1^ orally by gavage^28^, and three V_A_-treated groups receiving low, medium, and high doses of V_A_ in corn oil (1500, 3000, and 4500 IU/Kg/day, equivalent to approximately 450, 900, and 1351 µg/Kg/day, respectively)^15^. V_A_ and DON administration was initiated one week post-SCO initiation and was continued for three weeks. In addition, six normal healthy rats were included as a normal control group.

#### Blood and tissue sampling

At the end of the treatment period, rats were anesthetized using desflurane inhalation anesthesia (8% in oxygen) delivered via a precision vaporizer in an induction chamber until a deep level of anesthesia was achieved. Adequate depth of anesthesia was confirmed by the absence of the pedal withdrawal reflex. Blood samples were then collected retro-orbitally, after which animals were euthanized by cervical dislocation under deep anesthesia. Brains and livers were rapidly isolated, washed with ice-cold saline, and blotted dry for subsequent analyses. Appropriate parts of the isolated brains and livers were sectioned and kept in 10% neutral buffered formalin for H&E/Congo red staining and Masson’s trichrome staining, respectively, for histopathological examination. In addition, formalin-preserved brain tissues were used for immunohistochemical staining. The remaining parts of isolated brains and livers were stored at -80°C until performing ELISA, quantitative real-time PCR (qRT-PCR), and biochemical testing.

#### Determination of V_A_ content in liver and brain tissues

##### Assessing gene expression of RBP in both liver and brain tissues

Gene expression of RBP in brain and liver tissues was done by applying one step qRT-PCR technique. Briefly, RNA isolation was done using a Trizole plus RNA purification kit according to the manufacturer’s instructions**.** RNA concentration and purity were determined spectrophotometrically by measuring absorbances at 260 and 280 nm (A260/A280 ranging from 1.8 to 2.0 is equivalent to RNA purity of 90%-100%). Reverse transcription was done to generate cDNA using the high-capacity cDNA reverse transcription kit from Qiagen (Hilden, Germany) according to the provided instructions. Amplification of cDNA was done by thoroughly mixing the reaction mixture with a total volume of 25 μL. The reaction mixture consisted of Rotor-Gene RT mix (0.25 μL), Rotor-Gene SYBR Green RT-PCR master mix (12.5 μL), template RNA (1μL) and RNase-free water (6.5 μL). Primer sets are presented in Table 1. Acquisition of data was done using Rotor-Gene Q-Pure detection, software version 2.1.0 (build 9); Qiagen, Hilden, Germany. Relative target to reference genes expression in samples was quantified and expressed as a normalized ratio using the ΔΔCt method by calculating the threshold cycles (Ct) values of target genes relative to reference genes^15^.

**Table 1:** Used Oligonucleotide primers for RT-PCR.

Primer		Sequence	Accession number
RBP1	Forward	5’- TTCAACGGGTACTGGAAGAT-3’	NM_012733.5
	Reverse	5’- TCCTGCACGATCTCTTTGTC-3’	
SOX-11	Forward	5’- TGTCTCAAGGTAGTTGCACA -3’	NM_053349.2
	Reverse	5’- CAGACTTCAAAGAGCCACGA -3’	
NGN2	Forward	5’- TGCTCTATTCCCATTGCTGT-3’	NM_001398677
	Reverse	5’- GCCCACGTAGAAAGAGATGA-3’	
β-actin	Forward	5’- ATCATTGCTCCTCCTGAGCG-3’	NM_031144.3
	Reverse	5’- GGACAATAGGCATTGTGACG-3’	

##### Determining ALDH1A1 level (responsible for V_A_ activation) in brain tissue by ELISA

ALDH1A1 levels (ng/mg protein) were determined using specific rat ELISA kit in the supernatants obtained from centrifuging 20% hippocampal tissue homogenates in PBS (pH 7.4) using a cooling-centrifuge (Centurion-scientific-K3series, UK) at a speed of 15,000 rpm and temperature of 5°C for 5 min. ELISA technique procedure was done according to the manufacturer’s instructions. The protein content in brain tissue was determined by applying the Bradford method^29^.

#### Influence of V_A_ on neurogenesis and AD pathology

##### Determining the level of the neurogenesis marker, DCX, in brain tissue by ELISA

Tissue preparation procedure for determining DCX levels (ng/mg protein) was similar to that mentioned in Sect. (2.3.5.2) and DCX levels were determined following the provided ELISA kit instructions.

##### Assessment of NGN2 and SOX-11 gene expression associated with neuronal differentiation by qRT-PCR

Gene expression analysis was performed following the same protocol described previously in Section "Assessing gene expression of RBP in both liver and brain tissues". The reaction employed Primer Assays containing the forward primers together with the miScript SYBR Green PCR Kit (Qiagen, Germany). This kit provides the miScript Universal Primer as the reverse primer along with the QuantiTect SYBR Green PCR Master Mix. Expression levels were normalized using RNU6-2 as the internal reference gene.

##### Assessment of the influence of V_A_ on SIRT-1 by immunohistochemistry

Formalin-fixed brain tissue sections were mounted on coated slides and subjected to the avidin–biotin complex (ABC) immunohistochemical method using an anti- SIRT-1 antibody^24^. SIRT-1 immunostaining was assessed in selected brain regions relevant to AD pathology, including the dentate gyrus of the hippocampus, cerebral cortex, and cerebellar gray matter. Microscopic examination was performed using a light microscope (Inverted Microscope ECLIPSE Ti-S, Nikon, Japan), ), and positive immunoreactivity appeared as brown nuclear staining. Both qualitative histological evaluation and semi-quantitative analysis of staining intensity were performed using ImageJ software by an investigator blinded to the experimental groups.

##### Evaluation of V_A_ efficacy on cholinergic neurotransmission

Choline, ACh concentrations (µmol mg^-1^ protein), choline acetyltransferase (U gm^-1^ tissue), and Ach esterase (U mg^-1^ protein) were assayed in the hippocampi colorimetrically using reagents provided by specific colorimetric assay kits following steps and instructions provided by the kits’ suppliers.

##### Determination of AD serum marker; tau protein level

Serum tau level was measured by a rat-specific ELISA kit according to the manufacturer’s instructions. Levels were assayed in the serum obtained following centrifugation of coagulated blood at 4,000 rpm for 15 min.

##### Histopathological examination of the hippocampal deposited Aβ and degenerated brain tissue

Ascending grades of alcohol (70–100%) were used to dehydrate formalin-fixed brain tissue samples. Samples were then paraffin-embedded and sectioned to prepare 3–4 μm-thick sections using a microtome. After that, sections were deparaffinized using two changes of xylene and rehydrated through descending grades of alcohol and brought into water. The prepared sections were stained using H&E or Congo red stain for detection of amyloid deposits^30^^,^ then dried and mounted in Canada balsam for microscopic examination using a light microscope (Inverted Microscope ECLIPSE Ti-S, Nikon, Japan).

The mean amount of Aβ protein aggregates in the hippocampi was quantified by analyzing images captured using ImageJ software. In addition, neuronal degeneration was indicated by the quantitative morphometric analysis of the pathological changes in the frontal cortex using ImageJ software. These include calculating the percentage of damaged neurons (defined by intensely stained nuclei, cytoplasmic vacuolation, or cell shrinkage). Sections were scored for degeneration using a scale of 0–4 in which 0 = no damage, 1: ≤ 25% damage, 2: 25%–50% damage, 3: 50%–75% damage, and 4: > 75% damage^15,31^.

For both H&E and Congo red-stained sections, images were captured from eight different fields for each rat from each group under the same magnification by an investigator blinded to the examined groups. All images were captured from comparable anatomical regions under the same magnification to ensure consistency in quantification.

### Association between AD and liver fibrosis

#### Histological evaluation of liver fibrosis, steatosis, and hepatocellular damage

##### Liver fibrosis grading and evaluation

For histopathological evaluation of liver fibrosis, formalin-fixed liver tissues were dehydrated, paraffin-embedded, and cut into 3–4 μm sections. These sections were deparaffinized and rehydrated using descending grades of alcohol. This was followed by Masson’s trichrome staining, microscopic examination using a light microscope (Inverted Microscope ECLIPSE Ti-S, Nikon, Japan) by an investigator blinded to the experimental group, and quantification of the blue-stained area, representing fibrous tissue content, in each section using ImageJ software. In addition, each section was scored according to the method of Ishak scoring system, which comprises 7 stages ranging from 0 to 6^24^.

##### Liver steatosis and hepatocellular damage grading and evaluation

Liver steatosis was histologically graded and expressed as the percentage of hepatocytes containing macrovesicular or microvesicular fat. Steatosis was scored on a scale of 0–3 as follows: 0: no fat, 1: up to 33% fat, 2: 33%-66% fat, and 3: > 66% fat.

Ballooning degeneration of hepatocytes (indicative of hepatocellular injury) was characterized by the presence of swollen hepatocytes with rarefied cytoplasm. The mean percentage of ballooned hepatocytes was calculated for each group and given scores ranging from 0 to 5, where 0: no ballooning, 1: < 5%, 2: 5–10%, 3: 10–20%, 4: 20–50%, and 5: > 50%. Quantification was performed in eight different fields per rat under × 200 magnification. All images were captured from comparable anatomical regions under identical conditions.

### Quantitative determination of hepatic levels of TGF-ꞵ and hydroxyproline by ELISA

TGF-ꞵ and hydroxyproline levels were measured in liver tissues using ELISA kits following the steps provided by their manufacturers’ instructions. Before assessments, liver samples were homogenized in PBS (pH 7.4) to prepare 20% homogenates using a cooling-centrifuge (Centurion-scientific-K3series, UK) adjusted at a speed of 15,000 rpm and temperature of 5°C for 5 min. Supernatants were collected and used to assay TGF-ꞵ and hydroxyproline levels^15,32^.

### Statistical analysis of the data

Data were analyzed using the IBM SPSS software package version 20.0*.* (Armonk, NY: IBM Corp). The normality of distribution was verified using the Shapiro–Wilk test. Quantitative data were expressed as mean ± standard deviation (SD). Comparisons among the different groups of normally distributed quantitative data were done using analysis of variance (ANOVA; F test) and were followed by a Post Hoc test (Tukey) for pairwise comparison. The significance of the results was judged at the 5% level^33^.

## Results

### V_A_ biodistribution in rats with induced AD following oral and intraperitoneal administration

Tracing the biodistribution of V_A_ was performed after repeated oral or i.p. administration in rats with induced AD. V_A_ -associated fluorescence was semi-quantified in frozen sections from different organs using confocal microscopy. Regarding the distribution following multiple oral dosing, considerable fluorescence was observed in brain and small intestine tissue sections, with no statistically significant difference between their mean fluorescence intensities (*p* = 0.997), while both were significantly heigher than those detected in the other examined organs (*p* < 0.001). This pattern may reflect the role of the small intestine in V_A_ absorption and early distribution following oral administration. Liver sections showed the next highest fluorescence intensity after brain and small intestine, significantly exceeding all remaining organs (*p* < 0.001, possibly related to its function as a primary V_A_ storage organ.

On the other hand, a different V_A_ biodistribution pattern was observed following multiple i.p. doses; the greatest fluorescence intensity was observed in sections obtained from brain tissue, which was significantly higher than any other organ (*p* < 0.001). This may suggest preferential distribution of systemically administered V_A_ toward neural tissue. The other organ with significantly high fluorescent intensity was the liver, with a mean fluorescence intensity significantly higher than those obtained from other organs (*p* < 0.001).

**Fig. 1:**
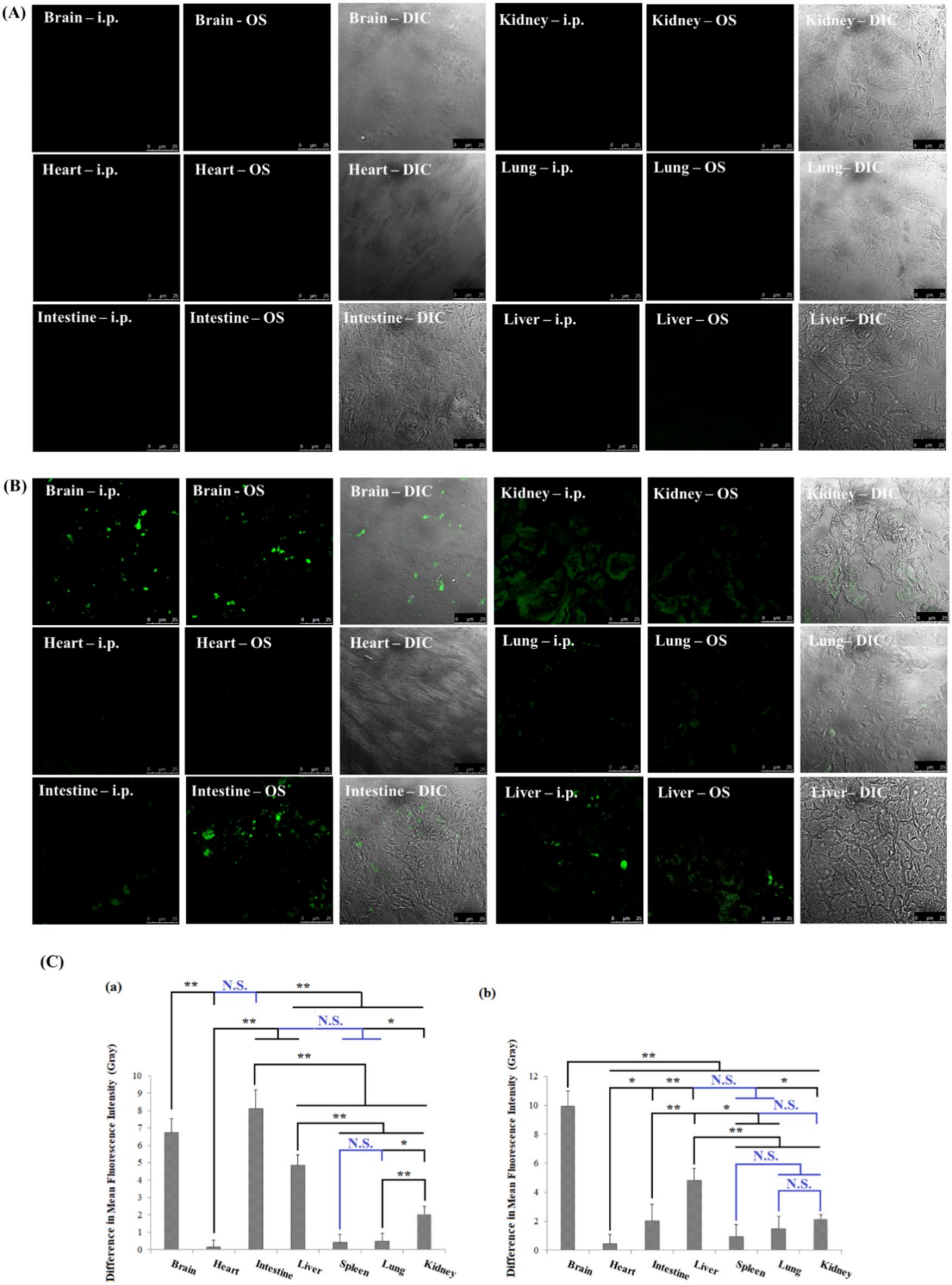
Biodistribution of V_A_ in rats with induced AD after oral or intraperitoneal (i.p.) administration, visualized by confocal laser microscopy. (**A**&**B**) Representative confocal micrographs of frozen sections from different organs of AD-induced rats before V_A_ treatment and following repeated V_A_ administration respectively. (**C**) Quantitative differences in the mean fluorescence intensity, expressed as (post-V_A_ administration − pre-V_A_ administration) for: (**a**) oral administration and (**b**) i.p. administration. Brain and small intestine showed the greatest fluorescence after oral dosing, whereas brain showed the highest fluorescence after i.p. administration. Tissue sections were analyzed using a confocal microscope (Leica DMi8, Wetzlar, Germany). Fluorescence images corresponding to V_A_ were captured at an emission wavelength (λem) of 695 nm, and green fluorescence intensity was semi-quantitatively analyzed using ImageJ software. (AD: Alzheimer’s disease; VA: vitamin A).

Sections obtained from hearts, lungs, and kidneys of rats with induced AD receiving either oral or i.p. V_A_ revealed comparable fluorescence intensities with no statistically significant difference between these organs (*p* > 0.05), Fig. 1. Collectively, these findings indicate route-dependent differences in V_A_ biodistribution across organs.

### Assessment of V_A_ association with α-SMA-positive reactive astrocyte-like cells in AD

**Fig. 2:**
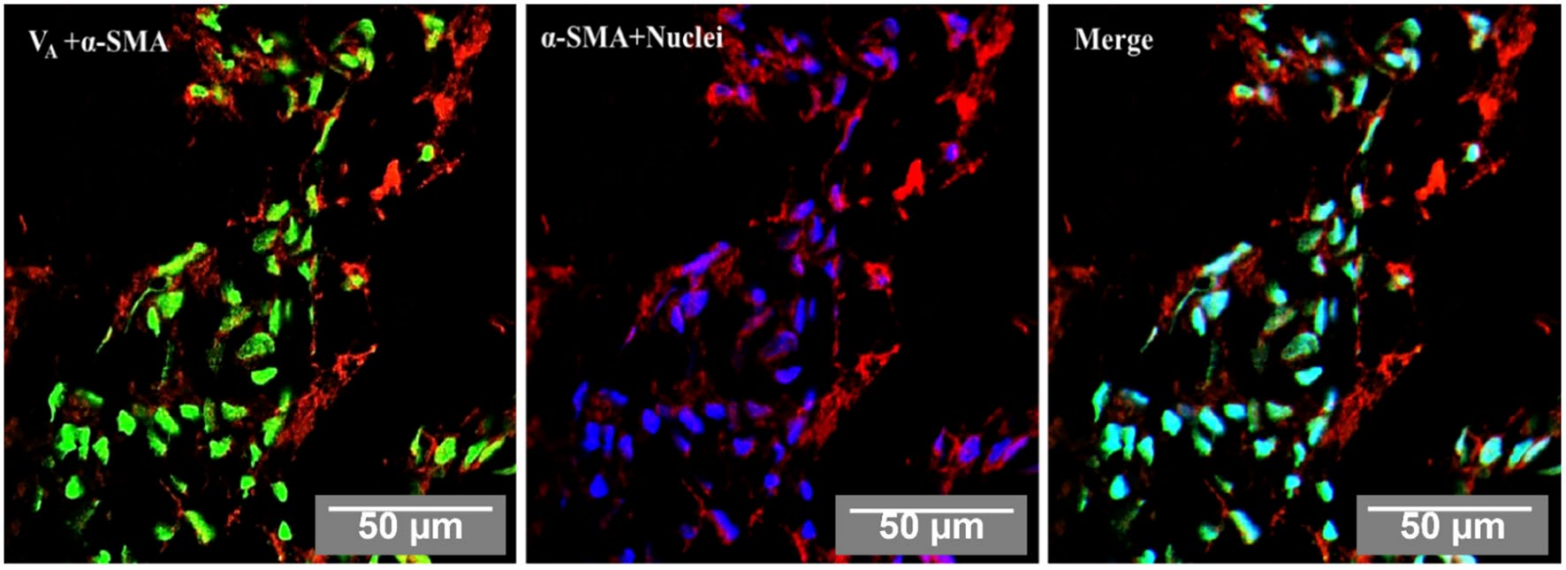
Confocal micrographs showing the spatial association of V_A_ fluorescence with α-SMA–positive reactive astrocyte-like cells in brain tissue of AD-induced rats. V_A_ signals are shown in green, α-SMA–positive cells in red, and nuclei stained with Hoechst in blue. Merged images demonstrate partial co-localization of V_A_ fluorescence with cell nuclei and spatial proximity to α-SMA–positive cells, indicating an association with cells exhibiting a reactive phenotype. Scale bar = 50 µm. All images were obtained from comparable anatomical regions of the hippocampus across experimental groups.

Figure 2 illustrates the spatial distribution of V_A_ signals in relation to α-SMA–positive reactive cells within brain tissue sections using confocal microscopy. V_A_ fluorescence (green) appeared predominantly localized within cellular nuclei, as indicated by overlap with Hoechst nuclear staining (blue). α-SMA–positive cells (red) were observed in proximity to these regions, and merged images demonstrated partial spatial overlap between V_A_ signals and α-SMA immunoreactivity. These findings suggest a potential association between V_A_ localization and cells exhibiting a reactive phenotype. However, α-SMA is not a lineage-specific astrocyte marker and may also label vascular smooth muscle cells, pericytes, or other mesenchymal-like reactive cell populations. Therefore, the observed co-localization should be interpreted as an association with α-SMA–positive reactive cells rather than definitive astrocyte-specific localization.

### Assessment of RBP in liver and brain tissues and ALDH1A1 in brain tissue

**Fig. 3:**
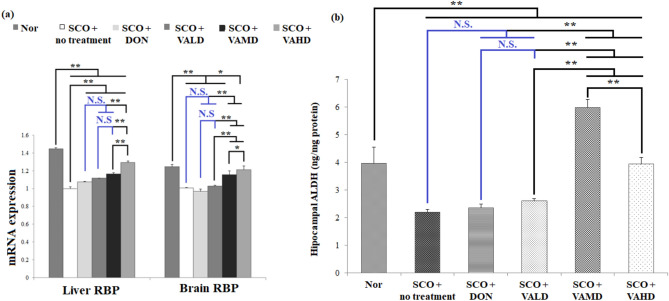
Effects of different treatments on (**a**) RBP expression (liver and brain) and (**b**) ALDH1A1 levels in brain tissue in AD-induced rats. (**a**) Both hepatic and brain RBP expression were reduced in untreated AD rats compared with normal controls, with a greater reduction observed in liver tissue. All treatments increased hepatic RBP expression, whereas only the VAMD and VAHD significantly increased brain RBP expression. The highest hepatic and brain RBP expression levels were observed in the VAHD group. (**b**) ALDH1A1 levels were reduced in untreated AD rats compared with normal controls and were not significantly altered by DON. V_A_ treatment increased ALDH1A1 levels, with the VAMD showing the highest level, significantly exceeding all treatment groups and normal controls. Data are presented as mean ± SD (n = 6). *p ≤ 0.05; **p ≤ 0.001; N.S., not significant.

In normal rats, the gene expression of hepatic RBP was significantly higher than brain RBP (*p* ≤ 0.001). Both hepatic and brain RBP gene expression levels in normal rats were significantly higher than those with SCO-induced AD (*p* ≤ 0.001), with a greater percentage reduction in expression level observed with hepatic RBP. In liver tissue, all tested therapeutics significantly increased RBP expression (*p* ≤ 0.001). In contrast, only VAMD and VAHD significantly increased brain RBP expression in brain tissue compared to the untreated rats with AD (*p* ≤ 0.001). VAHD therapy resulted in the highest hepatic/brain RBP gene expressions, which were significantly higher than those obtained with the other treated groups (*p* ≤ 0.05), Fig. 3a. This may indicate increased V_A_ availability at higher doses.

ALDH1A1 is responsible for V_A_ conversion into the active form RA. Significantly higher levels were detected in the hippocampi of normal rats as compared to rats with SCO-induced AD (p ≤ 0.001). DON had no effect on ALDH1A1 levels with no statistically significant difference compared to the untreated rats with induced AD (p > 0.05). Among the tested V_A_ doses, the medium dose produced the highest ALDH1A1 levels, which was significantly higher than all experimental groups even the normal group (p ≤ 0.001), Fig. 3b. This finding may suggest more efficient conversion of V_A_ to its active form at the medium dose.

### Influence of V_A_ on the brain levels of the neurogenesis marker, DCX

**Fig. 4:**
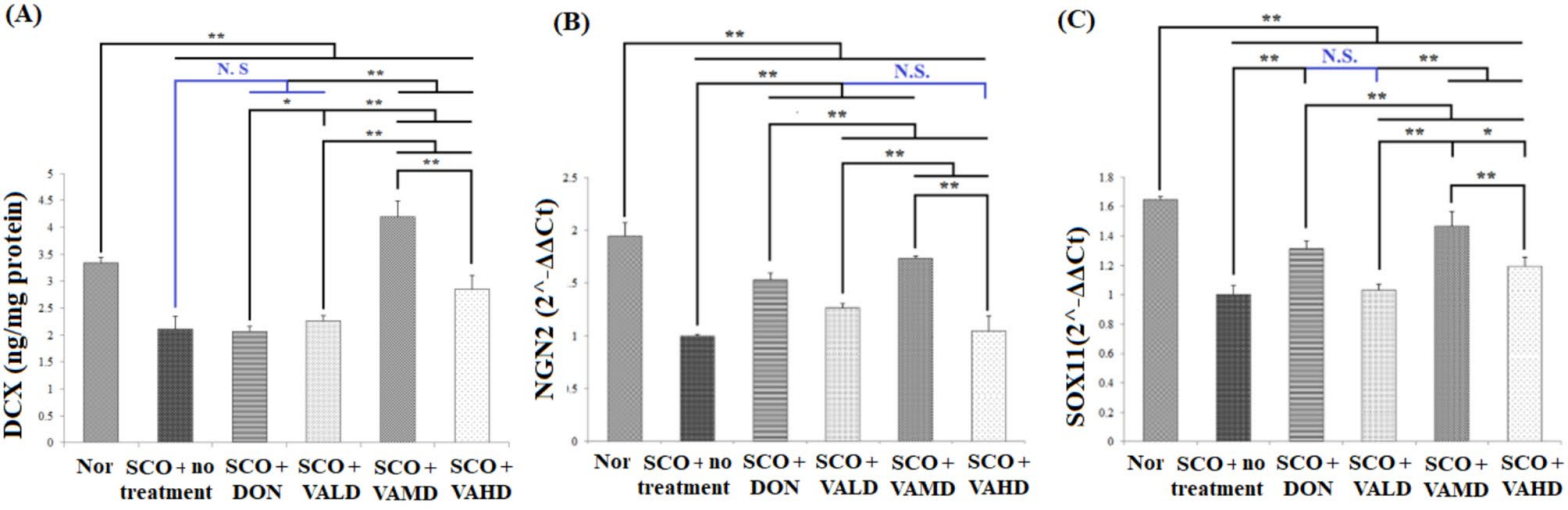
Effects of experimental treatments on: (**A**) DCX, (**B&C**) NGN2, and SOX-11 in rats with SCO-induced AD. (**A**) DCX levels were reduced in untreated AD rats, with the greatest increase observed in the VAMD group. (**B,C**) NGN2 and SOX-11 expression were reduced by SCO-induced AD and improved by treatment, except in the VAHD and VALD groups, which did not show significant increases in NGN2 and SOX-11 expression, respectively with VAMD showing the highest expression. Data are presented as mean ± SD (n = 6). * p ≤ 0.05; ** p ≤ 0.001; N.S., not significant.

SCO-induced AD significantly decreased the hippocampal levels of DCX compared with normal controls (*p* < 0.001). DON and VALD treatments showed no significant difference in DCX levels compared to the positive control group (*p* > 0.05), suggesting limited effect of these treatments on this marker. whereas VAMD treatment produced significantly higher DCX levels than all other experimental groups, including normal controls , Fig. 4A^,^ which may indicate that the medium dose exerts a more pronounced effect on neurogenesis-associated activity compared to other doses.

### Assessment of NGN2 and SOX-11 gene expression associated with neuronal differentiation by qRT-PCR

SCO-induced AD significantly reduced SOX-11 and NGN2 expression in brain tissue compared with normal controls. All treatments significantly increased NGN2 and SOX-11 expressions compared to the positive control group (*p* ≤0.001), except the VAHD and VALD groups, which did not show significant increases in NGN2 and SOX-11 gene expression, respectively (*p* >0.05). Among treatment groups, the VAMD-treated group revealed the greatest expression of both transcription factors, which was significantly higher than other treatment groups (*p* ≤0.001), Fig. 4 B &C. This may reflect enhanced activation of transcription factors associated with neuronal differentiation.

### Influence of V_A_ on SIRT-1 immunostaining; a neuroprotective HDAC and a regulator of AD’s TFs

**Fig. 5:**
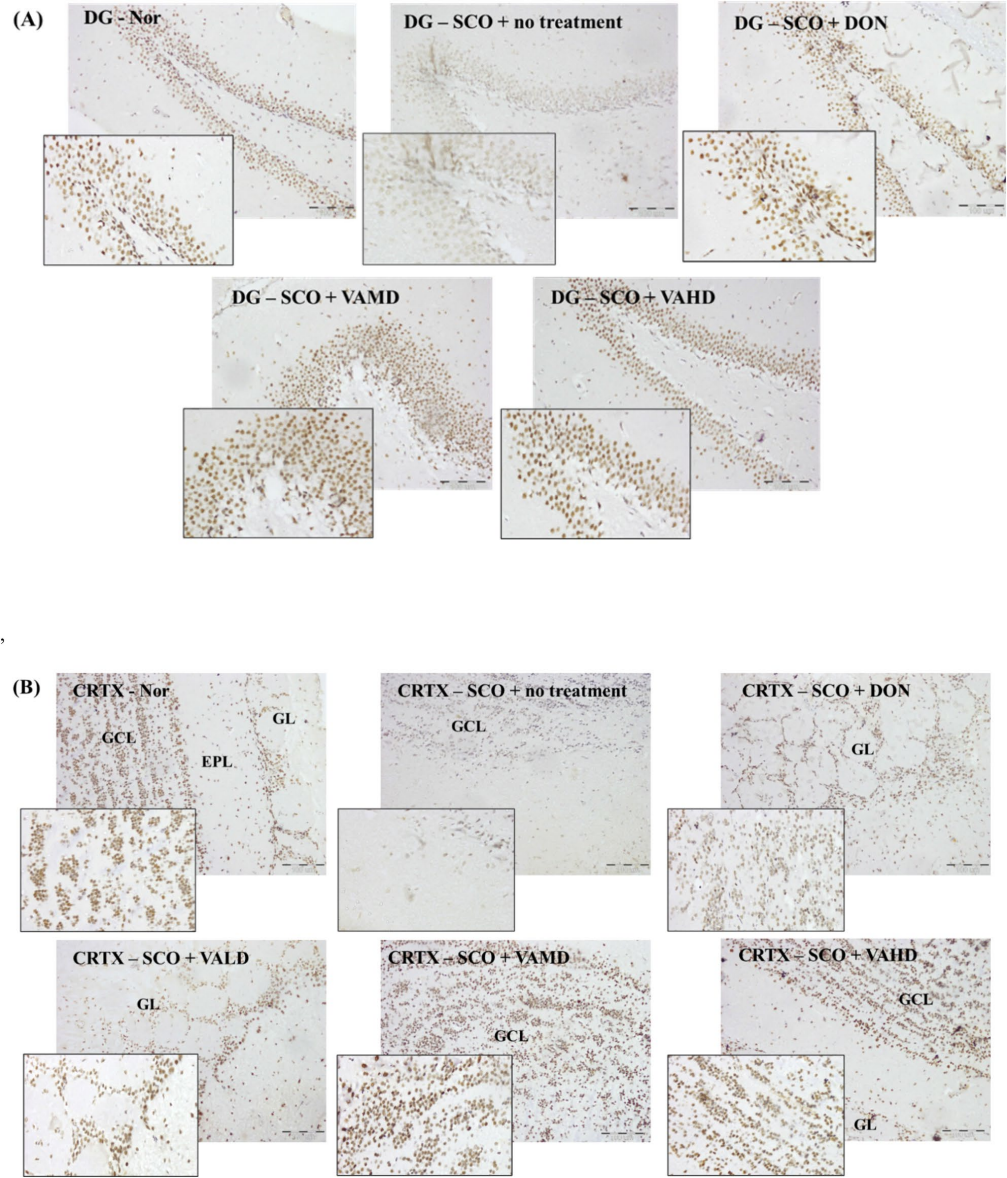

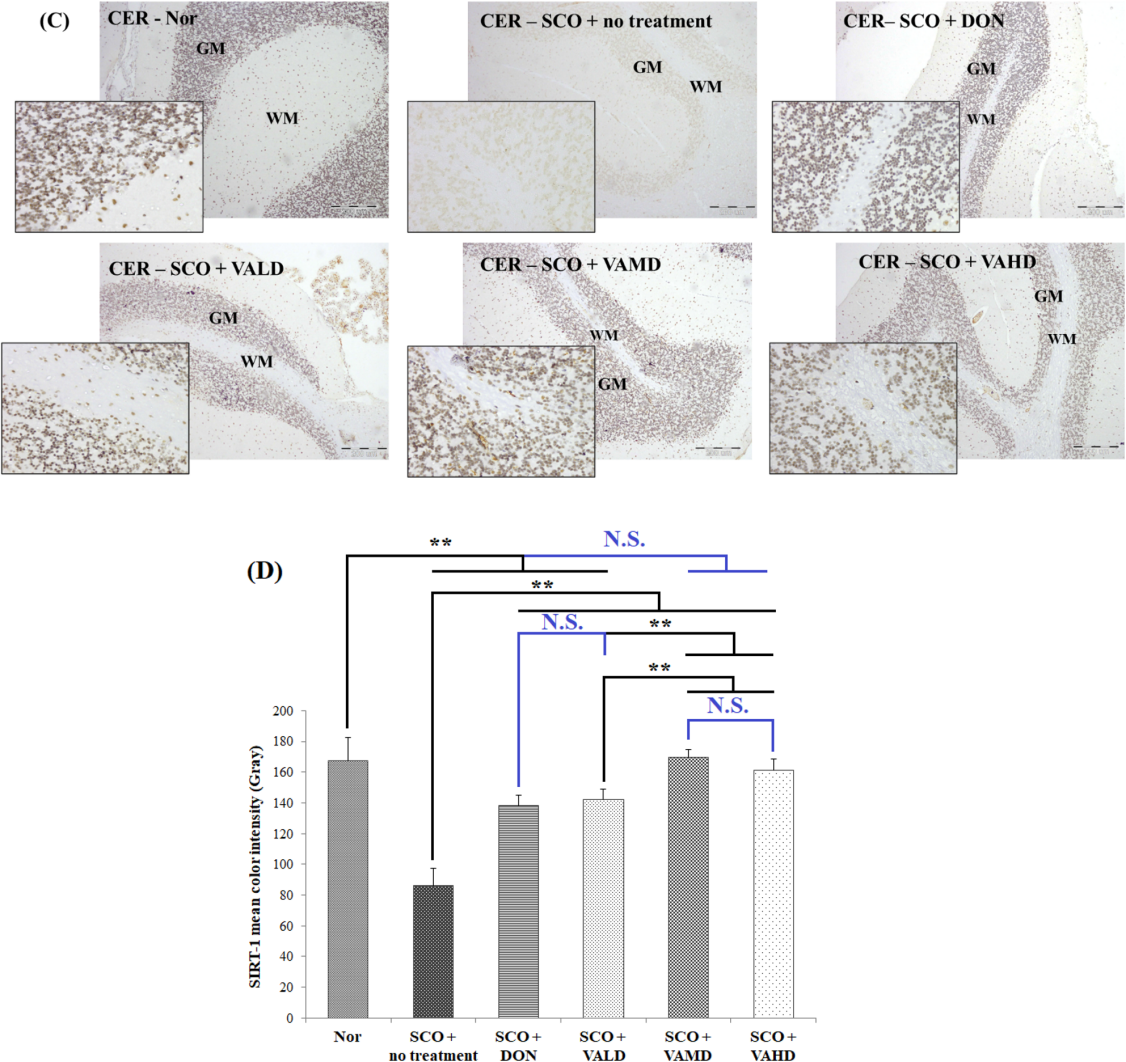
Effects of experimental treatments on SIRT-1 immunostaining in different brain regions of rats with SCO-induced AD. (**A**) Representative immunohistochemical micrographs showing nuclear SIRT-1 staining in the dentate gyrus (DG). (**B**) SIRT-1 immunostaining in the cerebral cortex (CRTX), central olfactory structures. (**C**) SIRT-1 immunostaining in the cerebellum (CER). (**D**) Semi-quantitative analysis of SIRT-1 immunostaining intensity. SCO-induced AD reduced SIRT-1 immunostaining in all examined brain regions, whereas treatment restored staining intensity, with VAMD and VAHD showing levels comparable to normal controls. Brown nuclear staining indicates positive immunoreactivity. All images were obtained from comparable regions across experimental groups under identical magnification and acquisition conditions. Data in (**D**) are presented as mean ± SD (n = 6). *p ≤ 0.05; **p ≤ 0.001; N.S., not significant. A and B: Scale bar = 100 µm, with higher-magnification insets (50 µm). C: Scale bar = 200 µm, with higher-magnification insets (50 µm). GM: gray matter, WB: White matter, GL: glomerular layer; EPL: external plexiform layer, GCL: granular cell layer.

In the present study, in brain sections obtained from normal rats, intense SIRT-1 nuclear immunostaining was observed in different brain areas, including the hippocampal, cerebral cortex, and cerebellum gray matter (Fig. 5A, B &C). In the hippocampi, nuclei of dentate gyrus neurons showed prominent staining. Within the cerebral cortex, the central olfactory structures were the most stained structures, where the nuclei at the granular cell layer showed intense staining, followed by the glomerular layer, and scarce staining was observed at the external plexiform layer. Within cerebellum tissues, the neuronal nuclei in the gray matter showed significant SIRT-1 staining. The induction of AD with SCO resulted in a significant reduction in the intensity of SIRT-1 immunostaining in different brain areas compared to that of the normal, undiseased rats (*p* ≤ 0.001). V_A_ and DON therapies significantly increased SIRT-1 expression compared to the positive control, untreated group (*p* ≤ 0.001). Notably, SIRT-1 expression in brain tissues derived from VAMD and VAHD-treated groups was comparable to normal, with no statistically significant difference observed between the two groups (p = 0.359), Fig. 5D, suggesting restoration of SIRT-1–associated signaling pathways.

### Influence of V_A_ on gene expression of endogenous transcription factors capable of reprogramming brain fibroblasts; SOX-11 and NGN2

SCO-induced AD significantly reduced SOX-11 and NGN2 expression in brain tissue compared to normal brain tissue. All treatments significantly increased SOX-11 and NGN2 expressions compared to the positive control group (*p*≤0.001), except for VAHD and VALD-treated groups, which failed to significantly increase NGN2 and SOX-11 gene expression, respectively (_p_>0.05). Among treatment groups, the VAMD-treated group revealed the greatest expression, which was significantly higher than other treatment groups (*p*≤0.001), Fig. 4 B &C. These findings suggest that V_A_ may enhance endogenous cholinergic neuroregenerative pathways, particularly at the medium dose.

### Evaluation of V_A_ influence on ACh synthesis and cholinergic neurotransmission

**Fig. 6:**
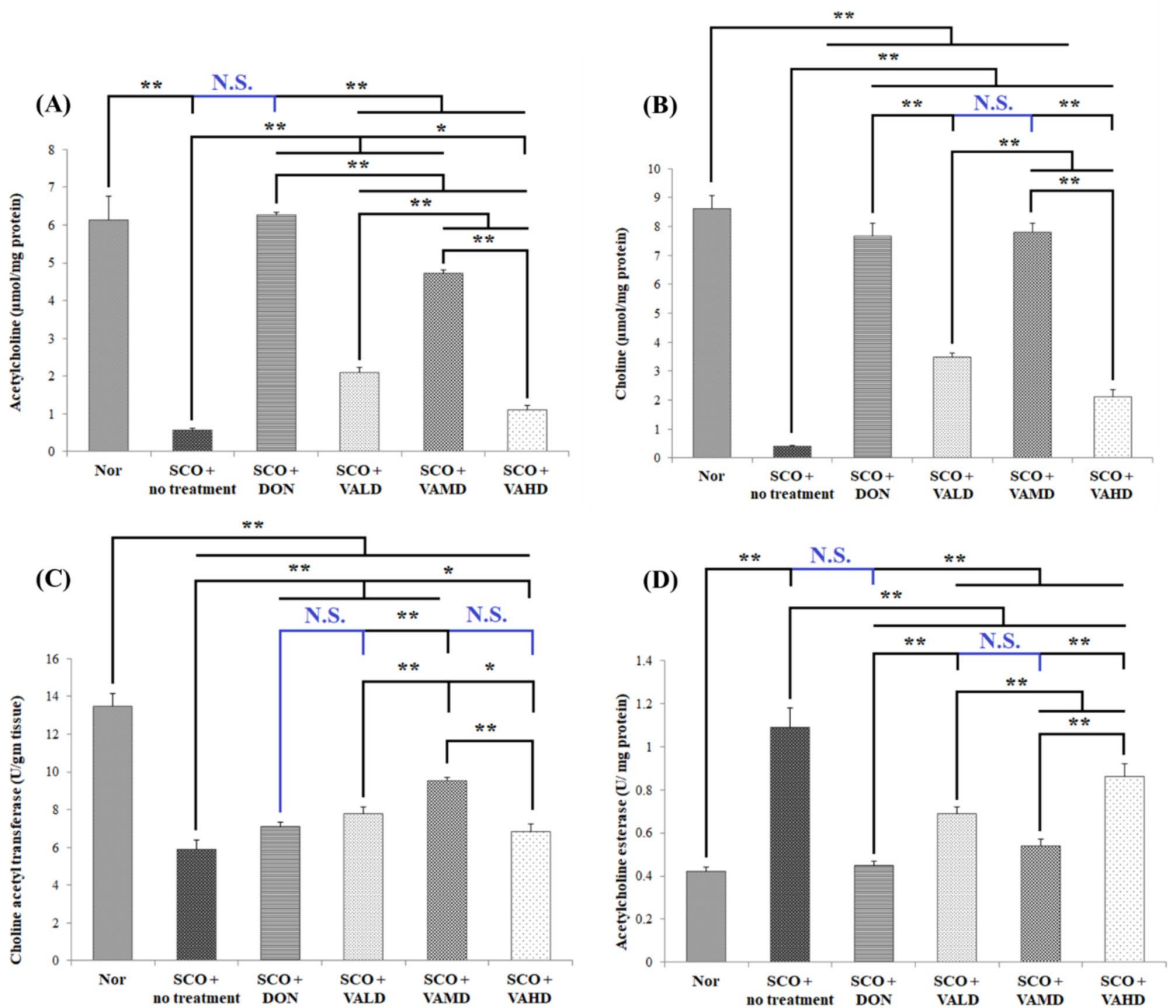
Effects of experimental treatments on cholinergic neurotransmission markers **A**: ACh, **B**: Choline, **C**: Choline acetyltransferase, **D**: Acetylcholinesterase. SCO-induced AD impaired cholinergic neurotransmission, evidenced by reduced ACh, choline, and choline acetyltransferase and increased acetylcholinesterase. Treatments attenuated these changes, with DON and VAMD showing the strongest restorative effects. Data are presented as mean ± SD (n = 6). * p ≤ 0.05; ** p ≤ 0.001; N.S., not significant.

Induction of AD significantly decreased brain levels of ACh, choline, and choline acetyltransferase when compared to the normal rats (*p* ≤ 0.001). Percentage decrease of ACh, choline, and choline acetyltransferase levels in brain tissues after induction were 91.7, 94.1, and 51.1%, respectively. On the other hand, AD induction resulted in a significant rise in the brain levels of AchE (*p* ≤ 0.001). All treatments significantly increased the ACh, choline, and choline acetyltransferase levels and decreased AchE. Amongst all treatment groups, DON and VAMD showed the most prominent effects; the statistically significant greatest levels of Ach and choline acetyltransferase were observed in DON and VAMD-treated groups, respectively (*p* ≤ 0.001). This finding suggests improved cholinergic neurotransmission. There was no statistically significant difference between both groups in the brain levels of choline and AchE (*p* = 0.981 and 0.16, respectively), Fig. 6.

### Histopathological evaluation of cortical degenerated neurons

**Fig. 7:**
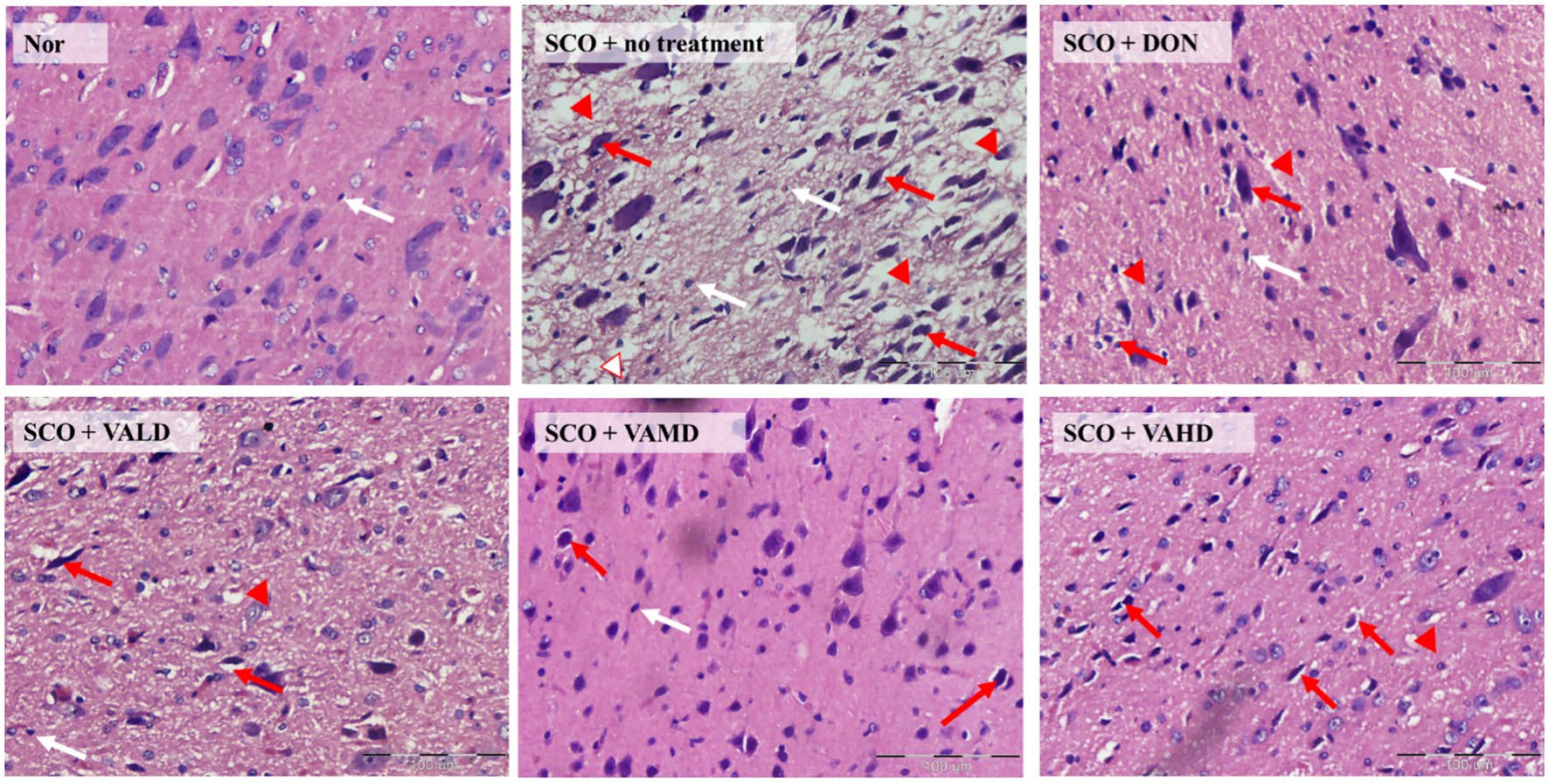
Representative H&E-stained cortical sections (× 200) from different experimental groups. Normal cortex (Nor) shows preserved neuronal morphology. SCO-induced AD sections (SCO + no treatment) display extensive neuronal degeneration (red arrows), shrunken dark neurons with pyknotic nuclei (white arrows), and cytoplasmic vacuolation (arrowheads). Treatment with DON, VALD, VAMD, and VAHD reduces neuronal damage to varying degrees, with the greatest improvement observed in the VAMD group. Scale bar = 100 µm.

The cortical sections of normal rats showed minimal neuronal degeneration with intact cytoplasmic architecture.. The degeneration scores for these sections ranged from 0-1 (degeneration percentage = 5.2±4.8%. The cortex samples with SCO-induced AD showed numerous shrunken, darkly stained degenerating neurons with pyknotic nuclei. All sections of rats’ brains with SCO-induced AD demonstrated extensivecytoplasmic degenerative changes and perivascular edema. The percentage of degenerated neurons in these sections was 94±9.8% (degeneration score=4). Treatment of rats with SCO-induced AD with either DON, VALD, VAMD, or VAHD significantly reduced the percentages of degenerated neurons, with degeneration scores ranging from 1–3 across treated groups, with the lowest score observed in VAMD-treated rats, Fig. 7 and Table 2.

**Table 2:** Percentage of degenerating neurons and degeneration score in the cortical tissue stained with H&E and quantified using ImageJ software.

Experimental group	Degenerating neurons%	Degeneration score
Nor	3.2 ± 2.8%	0–1
SCO + no treatment	94 ± 9.8%^a^	4
SCO + DON	19.3 ± 9.7% ^a,b^	1–2
SCO + VALD	45.7 ± 8.2% ^a,b,c^	2–3
SCO + VAMD	11.1 ± 3.7% ^a,b,c,d^	1
SCO + VAHD	33.9 ± 11.6% ^a,b,c,e^	2

The degeneration scale ranges from 0 to 4; 0: no damage, 1: ≤ 25% damage, 2: 25%–50% damage, 3: 50%–75% damage, and 4: > 75% damage. ANOVA test was used to compare the different groups with the Post Hoc Test (Tukey). a: Statistically significant difference between this group and Nor group, b: Statistically significant difference between this group and SCO + no treatment group, c: Statistically significant difference between this group and SCO + DON group, d: Statistically significant difference between this group and SCO + VALD group, e: Statistically significant difference between this group and SCO + VAMD group. (Nor: normal control, SCO: scopolamine, DON: donepezil, VALD: vitamin A-low dose, VAMD: vitamin A-medium dose, VAHD: vitamin A-high dose). Quantification was done for images captured from eight different fields for each rat from each group and examined under the same magnification power.

### Influence of treatments on hippocampal Aβ deposition and the AD serum marker tau

**Fig. 8:**
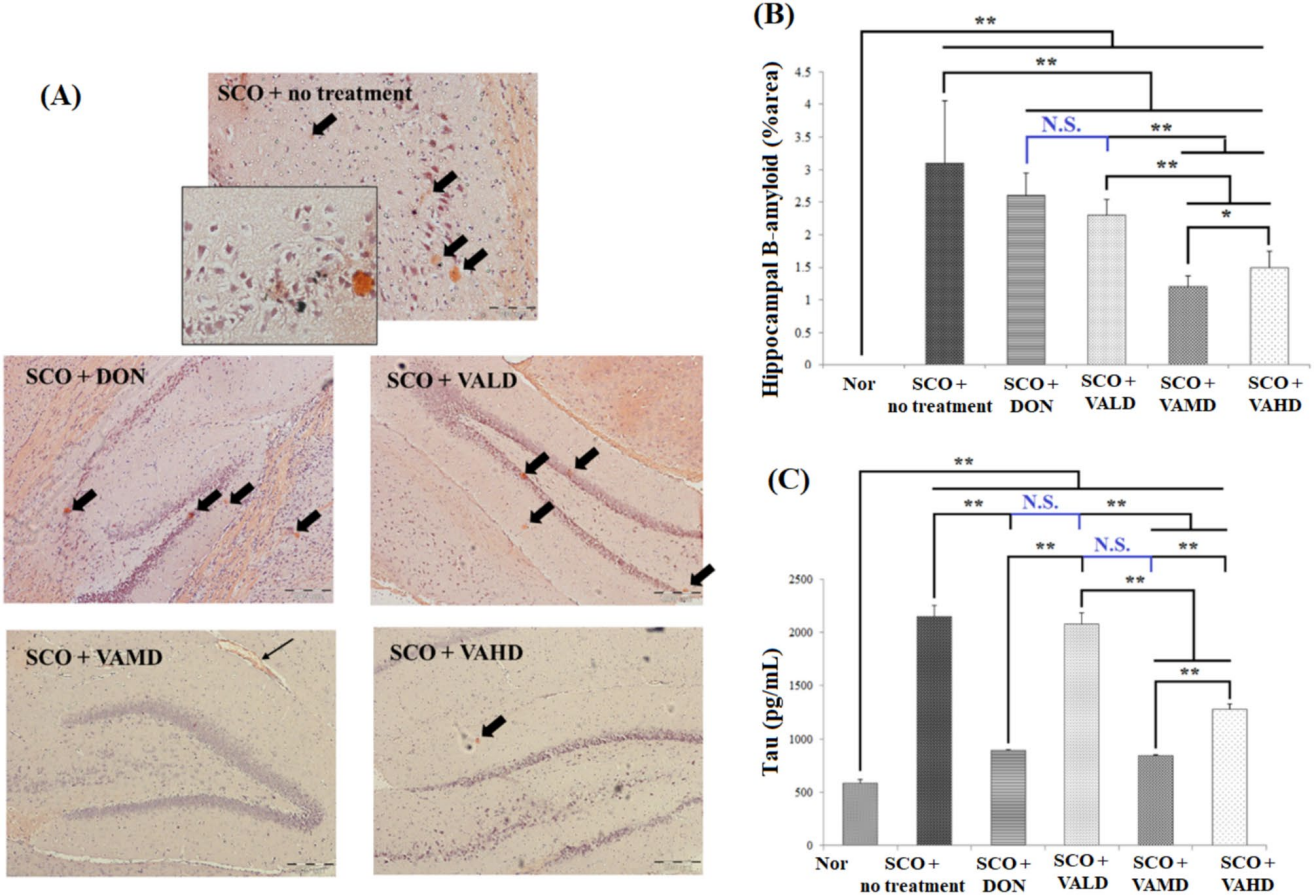
Representative Congo red-stained hippocampal sections of different groups and quantified Aβ deposition and Tau protein level. (**A**) Representative micrographs showing hippocampal Aβ deposits across experimental groups. (**B**) Quantification of deposited hippocampal Aβ. (**C**) Quantification of serum tau protein levels. SCO-induced AD increased Aβ deposition and serum tau levels, whereas treatments attenuated these changes, with VAMD showing the strongest reduction in Aβ deposition. Thick black arrows indicate parenchymal Aβ deposits, and thin black arrows indicate vascular Congo red staining. Magnification × 100; inset in the SCO + no treatment group × 400. Data are presented as mean ± SD (n = 6). * p ≤ 0.05; ** p ≤ 0.001; N.S., not significant.

The amount of deposited Aβ in the Congo red-stained hippocampi was semi-quantified using ImageJ software. In addition, the AD marker, tau protein, was assessed in serum using the ELISA technique. The untreated rats with SCO-induced AD showed significantly more deposited Aβ and higher serum tau protein levels (pg/mL) compared to the normal group (*p* ≤ 0.001). All treatments significantly decreased Aβ deposition and serum tau protein levels compared to the untreated rats with induced AD (*p* ≤ 0.001). VAMD showing the strongest reduction in Aβ deposition, suggesting a more pronounced effect on AD-related pathological markers. VALD, did not significantly reduce serum tau levels (*p* = 0.443). There was no statistically significant difference between DON and VAMD-treated groups in reducing serum tau protein levels (*p* = 0.773), whereas the DON-treated group showed significantly more deposited Aβ in the hippocampi (*p* ≤ 0.001), Fig. 8.

## Evaluation of the association between AD and liver fibrosis

### Histological evaluation of liver fibrosis, steatosis, and hepatocellular damage

Histological assessment of liver fibrosis was done by semi-quantitatively analyzing the amount of fibrous tissue (blue color) in Masson’s-trichrome-stained liver tissue sections using ImageJ software, grading the degree of fibrosis according to Ishac score, analyzing and scoring the percentages of steatosis and hepatocyte ballooning.

**Fig. 9:**
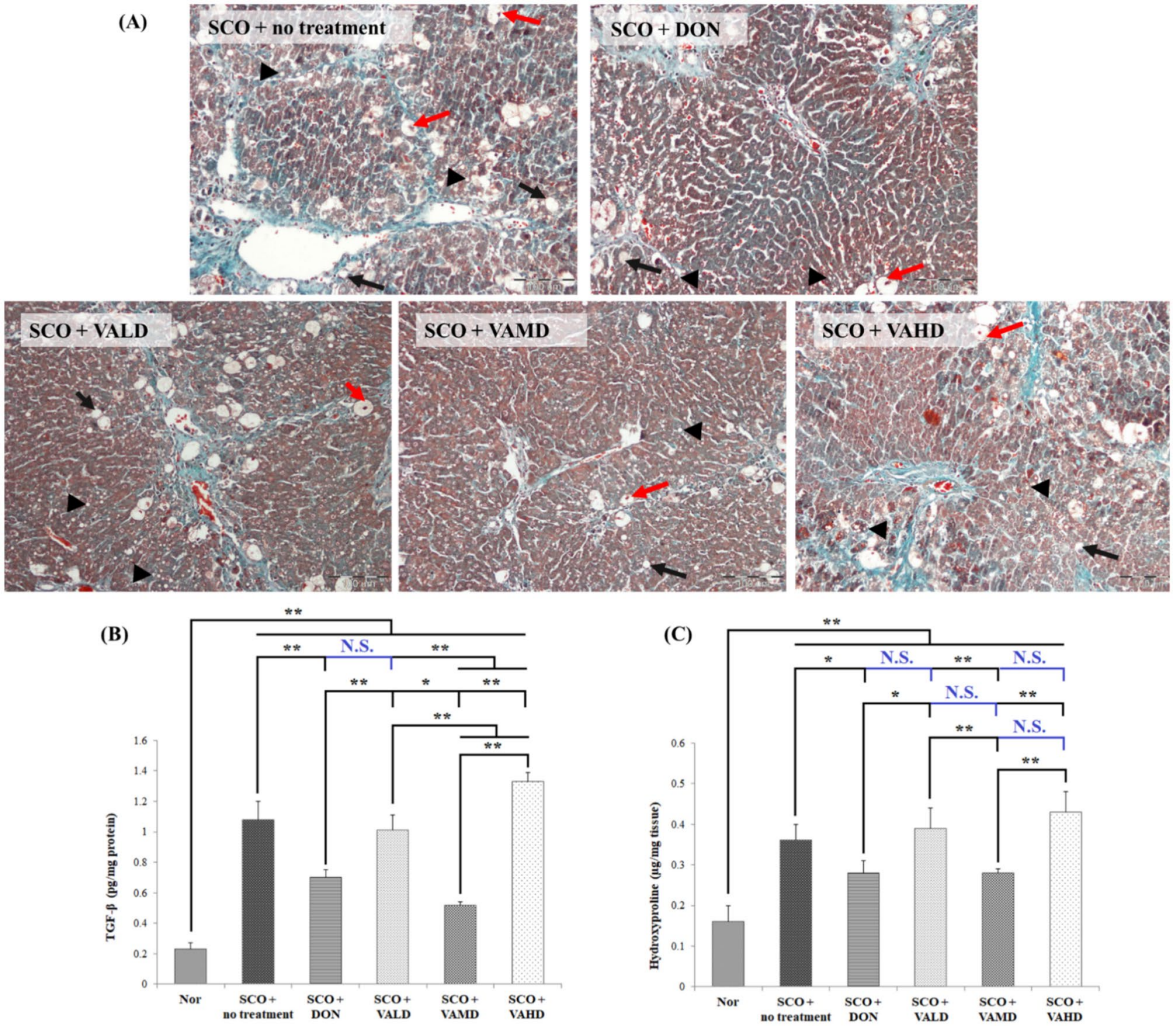
Effect of experimental treatments on liver fibrosis histologically and biochemically on rats with SCO-induced AD. **A**: Representative Masson’s trichrome-stained liver sections (× 200) showing fibrotic deposition (blue colour), macrovesicular steatosis (black arrows) and microvesicular steatosis (black arrowheads), and hepatocyte ballooning (red arrows ) across experimental groups. . **B**: Hepatic TGF-ꞵ levels (pg/mg protein) **C**: Hepatic hydroxyproline levels (μg/mg tissue). Treatments attenuated histological and biochemical markers of liver fibrosis, with the greatest improvement observed in the VAMD group. Data are presented as mean ± SD (n = 6). * *p* ≤ 0.05; ** p ≤ 0.001; ns, not significant.

Sections obtained from the untreated group with SCO-induced AD revealed marked fibrotic alterations, including bridging fibrosis and increased fibrous deposition (Ishak scores 3–4). Quantitative analysis revealed significantly greater fibrotic tissue content than in normal rats (*p* < 0.001). These sections also exhibited prominent hepatocyte ballooning and predominantly zone 3 macrovesicular and microvesicular steatosis (score 2).Liver sections obtained from treatment groups showed significantly reduced fibrotic deposition, steatosis, and hepatocyte ballooning.. The greatest histological improvement was observed in the VAMD-treated group, indicating attenuation of fibrosis-associated changes showing fibrous expansion of only some portal areas with or without short fibrous septa (Ishak scores 1), steatosis < 33% (11.5 ± 4.3% ) (score 1), and hepatocyte ballooning < 5% (2.2 ± 0.8%) (score 1), Fig. 9A and Table 3.

**Table 3:** Quantitative and semiquantitative assessment of liver fibrosis, steatosis, and hepatocyte ballooning in Masson’s trichrome-stained liver sections.

Experimental group	Fibrotic tissue intensity (gray value) (Ishak score)	Steatosis percentage (score)	Hepatocyte ballooning percentage (score)
Nor	113.1 ± 6.8 (0)	1.4 ± 0.4% (0)	0.2 ± 0.1 (0)
SCO + no treatment	171 ± 9.8^a^ (3–4)	52.2 ± 10.4%^a^ (2)	7.2 ± 2.1%^a^ (2)
SCO + DON	132.4 ± 7.3^a,b^ (1–2)	21.4 ± 3.7%^a,b^ (1)	3.1 ± 1.9%^a,b^ (1)
SCO + VALD	143.7 ± 6.2^a,b,c^ (2–3)	24.2 ± 4.6%^a,b^ (1)	4.1 ± 1.3%^a,b^ (1–2)
SCO + VAMD	121.1 ± 4.4^a,b,c,d^ (1)	11.5 ± 4.3%^a,b,c,d^ (1)	2.2 ± 0.8%^a,b,c,d^ (1)
SCO + VAHD	155.3 ± 10.2^a,b,c,d,e^ (2–3)	31.9 ± 5.7%^a,b,c,e^ (1–2)	5.3 ± 1.4%^a,b,c,e^ (1–2)

### Group comparisons were performed using one-way ANOVA followed by Tukey’s post hoc test

Superscripts indicate statistically significant differences:a: vs. Nor group, b: vs. SCO + no treatment group;, c: vs. SCO + DON group;, d: vs. SCO + VALD group; , e: vs. SCO + VAMD group. . Quantification was based on for images captured from eight different fields per rat at identical magnification from comparable anatomical regions.

### Influence on liver fibrosis markers; TGF-ꞵ and hydroxyproline

The mean TGF-β and hydroxyproline levels were significantly higher in untreated rats with SCO-induced AD compared to normal rats (*p* ≤ 0.001). Treatment with VALD and DON did not significantly reduce hepatic hydroxyproline concentrations relative to the untreated group (*p* > 0.05). VALD did not significantly reduce TGF-β levels. Among treatment groups, VAMD produced the greatest reduction in both hepatic TGF-β and hydroxyproline levels compared to untreated AD rats (*p* ≤ 0.001). However, all treated groups remained significantly higher than normal controls (*p* ≤ 0.001), Fig. 9 B&C. 

## Discussion

AD is the most common type of neurodegenerative diseases with multiple etiopathological theories, including amyloid angiopathy, tauopathy, cholinergic neuron degeneration, and failure of the brain’s cholinergic system^2^. The failure of the FDA-approved AD therapeutics surged great enthusiasm to find a cure for AD. Intensive research came to the assumption that the actual AD disease-modifying agent is the one capable of replenishing the degenerated cholinergic neurons.

The present investigation revealed several key findings relevant to the understanding of AD pathophysiology and potential therapeutic modulation by V_A_. First, brain VA deficiency was observed in AD, and both orally and systemically administered V_A_ showed preferential accumulation in brain tissue. Second: V_A_ signals were spatially associated with cells exhibiting a reactive phenotype in AD brain tissue. Third: V_A_, at a medium dose, promoted neurogenesis as evident by increasing DCX levels (a neurogenesis marker) and enhanced the brain’s cholinergic system (increased ACh synthesis and decreased its degradation). These effects may be partially mediated by the V_A_-induced overexpression of SIRT-1, an HDAC that modifies several TFs. Accordingly, VA increased the expression of neurogenesis-associated transcription factors, including SOX-11 and NGN2. Fourth, V_A_ significantly decreased AD markers, tau protein, and Aβ deposition in brain tissue. Lastly, SCO-induced AD was associated with hepatic V_A_ deficiency and hepatic fibrosis that were reversed by VAMD administration.

The current AD model employed in this study was based on chronic SCO administration to induce sustained cholinergic blockade. This model reproduces several features relevant to AD pathology, including neuronal degeneration, Aβ and tau accumulation, oxidative stress, neuroinflammation, and impaired cholinergic signaling. Accordingly, it is widely used to investigate AD-related mechanisms and to evaluate potential therapeutic interventions at cellular and molecular levels^34–36^. However, it does not fully recapitulate the complexity of human AD.

In the present study, V_A_ was administered at doses of 1500, 3000, and 4500 IU/kg/day to evaluate its dose-related effects. When translated to human equivalents, the low and medium doses fall within commonly used supplementation ranges, whereas the high dose approaches levels associated with potential toxicity. Although high-dose regimens may be used clinically for deficiency correction, prolonged exposure is linked to adverse effects, particularly hepatotoxicity^37,38^. In the current model, signs consistent with hepatotoxicity were observed at the highest V_A_ dose, supporting a dose-dependent safety concern. These findings highlight the importance of dose optimization in potential translational applications.

Brain astrocytes are part of the diffuse stellate cells (V_A_-storing cells) system, mimicking the HSCs structurally and functionally. These astrocytes become activated, matrix-producing cells responding to brain injury under pathological conditions^20,25^. Generally, stellate cells lose their VA-storing capacity upon activation. However, it is not clear whether this could happen to activated brain astrocytes. A key consideration is that astrocytes are integral to neural homeostasis and Aβ plaque regulation,thus, their modulation may have dual effects, and excessive alteration could potentially result in functional imbalance.

Previous work from our group reported that astrocyte activation in a haloperidol-induced PD model is associated with reduced V_A_ levels in brain tissue, and that VA supplementation selectively targets the V_A_-deficient brain astrocytes^15^. In the present study, we investigated whether a similar pattern occurs in AD. Accordingly, multiple VA doses were administered orally and intraperitoneally to rats with induced AD, and their biodistribution was assessed using confocal microscopy based on intrinsic V_A_ fluorescence. Oral administration resulted in prominent fluorescence in the brain and small intestine, whereas intraperitoneal administration led to predominant accumulation in brain tissue. The liver also showed relatively high fluorescence following both routes of administration.

These findings are consistent with previous reports indicating reduced VA and β-carotene levels in AD patients, as well as impaired retinoid signaling, including reduced retinoic acid receptor expression and decreased activity of retinoid-synthesizing enzymes^19,39,40^.

More specifically, V_A_ fluorescence was observed in close spatial association with α-SMA–positive reactive cells in brain tissue, as demonstrated by confocal microscopy. These findings suggest that V_A_ accumulates in regions exhibiting a reactive cellular phenotype rather than confirming specific astrocytic localization.

As the activated brain astrocytes; is the main source of RA for the regulation of brain neurogenesis^26,41^, this findings suggests that these cells lose their V_A_ storage capacity in active AD and externally administered V_A_ can target activated astrocytes.

Notably, VA signals showed prominent nuclear localization, which may reflect engagement of retinoid-related signaling pathways, given that retinoic acid (RA) exerts its biological effects through nuclear receptors (RARs). Under oxidative stress conditions, retinaldehyde dehydrogenase translocates from the cytoplasm to the nucleus^27^. RAR activation by RA regulates neural progenitor and astrocytic differentiation into multiple neural cell types. Further, RA controls the action of TFs via epigenetic routes^27^.

The accumulation of V_A_ in the intestinal tissue following oral administration has been previously reported and was interpreted as the probable uptake of orally administered V_A_ by the intestinal stellate cells that act as a temporary VA storage site until directed toward VA-deficient sites (the brain in the AD case)^15,24^. The detectable VA accumulation in hepatic tissue, the main VA storage site, following either oral or i.p. administration indicates the probable existence of VA deficiency in liver tissue and more specifically within the HSCs that uptake some of the administered VA, but to a much lesser extent than did the brain astrocytes.

Comparison of hepatic and brain V_A_ status was assessed by measuring RBP gene expression in liver and brain tissues. Due to the lipophilic nature of retinoids, they are carried by specific proteins in the blood and inside cells. The RBP can be detected within the liver and brain cellular components. The presence of RBP within cells was previously elucidated by the occurrence of receptor-mediated uptake of the RBP-retinol complex^42–44^. Consistent with its role as a primary V_A_ storage site, hepatic RBP expression was higher than brain levels in normal rats. AD induction significantly reduced RBP expression in both tissues, , reflecting possible replenishment of the brain V_A_-deficient astrocytes by the stored V_A_ in HCSs to achieve equilibrium between V_A_-storage pools^24^. Thus, nearly equal brain and hepatic RBP gene expressions were detected.

V_A_ supplementation increased RBP expression in a dose-related manner, with the highest levels observed in the VAHD group. In contrast, ALDH1A1, which mediates conversion of V_A_ to its biologically active form (retinoic acid), showed a different pattern, with the highest levels detected in the VAMD group, exceeding normal controls.

These findings suggest that while higher doses may enhance V_A_ availability, the medium dose may more effectively promote its conversion to the active form, which may contribute to its more pronounced biological effects observed in this study.

The potential of activated astrocytes to contribute to neuroregenerative processes and support neuronal replacement strategies in AD remains an area of active investigation^9^. Exogenous TFs, NGN2 and SOX-11, previously managed to convert fibroblasts into efficient cholinergic neurons^12^. V_A_ is known for its regenerative capabilities, especially in neurodegeneration^16,17^. Herein, VAMD significantly increased the hippocampal levels of the neurogenesis marker, DCX, compared to other groups and even more than the baseline observed in the normal rats, indicating active neurogenesis. DCX is a reliable and specific marker that reflects neurogenesis and its modulation and can be used to study neurogenesis under normal and pathological conditions^45,46^. Further, the potential influence of VA on endogenous TFs associated with neuronal differentiation, including NGN2 and SOX-11, was evaluated.

NGN2 is a key transcription factor widely used to induce neuronal differentiation from various cell types, including stem cells, glial cells, and fibroblasts^47^. Previous studies have demonstrated that members of the SoxC family, particularly SOX-11, play a critical role in enhancing NGN2-driven neuronal differentiation^12,48–50^.

The interaction between NGN2 and SOX-11 has been shown to promote neurogenic transcriptional programs and support neuronal lineage commitment. In particular, co-expression of these factors enhances neuronal differentiation efficiency compared to NGN2 alone. These findings highlight the importance of NGN2/SOX-11 interplay in regulating neurogenesis-related pathways^51,52^.

In the present study, normal brain tissue showed higher expression of TFs associated with neuronal differentiation, including NGN2 and SOX-11. SCO-induced AD significantly reduced the expression of these TFs, indicating impaired neurogenesis-associated processes.Treatment with DON increased NGN2 and SOX-11 expression; however, the effect was less pronounced compared to VAMD. The biological effects of DON have been reported beyond AD, including roles in cellular differentiation and tissue repair^53,54^. However, in AD, its clinical benefit is generally limited to symptomatic improvement in cognitive function, without clear evidence of disease modification, and may be associated with adverse effects^55^. This limited efficacy is consistent with its primary mechanism as AChEI, which enhances synaptic ACh levels rather than directly modulating neurogenesis-related pathways.

V_A_ showed dose-selective rather than dose-dependent stimulation of TFs’ expression, with the greatest potential attained by the medium dose. VAMD therapy showed significantly higher gene expression levels of NGN2/SOX-11 compared to DON therapy. This dose selectivity of V_A_ to induce neuronal regeneration is in line with Prof. Maden’s conclusions about the effect of retinoids on amphibian limb regeneration^56^. He stated that cells will differentiate into a particular tissue using a certain morphogen’s concentration, and at a different morphogen concentration, cells will differentiate into another tissue^56^. Results of the present study designated VAMD as dose-specific for regenerating brain neurons. Other V_A_ doses may be more efficient for inducing regeneration in other organs. Similar results were obtained when testing the regenerative ability of V_A_ in dopaminergic neurons in PD, and VAMD showed the greatest regenerative potential^15^.

Retinoids have many distinctive functions in the brain, including neuronal differentiation, axonal outgrowth, and regeneration. Retinoids can induce the regeneration of axons after damage and the generation of specific neuronal cell types. They also maintain adult neurons and neural stem cells in the differentiated state. RA signaling is crucial for synaptic plasticity, learning, and memory, and the loss of RA signaling is involved in the etiology of AD^57,58^. Retinoids’ roles in the CNS are carried out through their interaction with retinoid receptors,RA receptors (RARα, β, and γ) and retinoid X receptors (RXRα, β, and γ). These receptors are extensively expressed in brain areas involved in AD pathogenesis, including the prefrontal cortex, hippocampus, and amygdala. Retinoids and their receptors are transcriptional regulators that modulate the transcription of many genes coding for many functional proteins, including TFs^57^. Therefore, activated V_A_ (RA) induced NGN2/SOX-11 gene expression after interaction with the retinoid receptors. In support of V_A_-induced NGN2/SOX-11 gene expression, the endogenous bioactive retinoids produced by the early embryonic neuroectodermal (NE-4C) stem cells induce more NGN2 expression to boost neuronal differentiation^59^. Further, in another study using Neuro2a neuroblastoma cells and cultured mouse dorsal root ganglia neurons, SOX-11 expression rose as these cells differentiated, and the addition of RA caused a further rise in SOX-11 level^60^. Moreover, in spinal motor neurons, a close connection was proved to exist between RA and NGN2,RAR binds to NGN2 to synergistically trigger transcriptionally active chromatin for cell fate specification^61^.

Data indicate that NGN2/SOX-11 expression is epigenetically regulated^10,13^. SIRT-1 is a highly conserved NAD + -dependent class III HDAC located inside the nucleus. SIRT-1 is one of the most studied HDACs in AD,those studies conclude that SIRT-1 overexpression exhibited protective effects on AD by mediating the survival of neurons and enhancing axonal growth, and synaptic plasticity through the deacetylation of target genes. On the other hand, SIRT-1 deficiency causes cognitive function impairment, neuronal degeneration, and excessive Aβ deposition^62,63^. HDACs/SIRT-1 tightly regulate the expression of NGN2 and SOX-11,For example, SIRT-1 activation by resveratrol promoted the neuronal differentiation and expression of the pro-neural TFs such as NGN2^63^^,^ and histone deacetylation induced SOX-11 expression during retinal development^64^. In the present investigation, SIRT-1 was immunostained in brain tissue and quantified in areas demonstrating extensive SIRT-1 positive staining. In brain tissues of normal rats, dentate gyrus neurons in the hippocampi, cerebral cortex (especially the central olfactory structures), and cerebellum gray matter showed prominent SIRT-1 immunostaining. As a neuroprotective protein, SIRT-1 is concentrated in the areas more susceptible to degenerative changes in the brain. Upon ageing, SIRT-1 was found to be located in cerebellar Purkinje cells, CA3 and CA4 regions of the hippocampus, neurons of the cerebral cortex, olfactory bulb, the dorsal root ganglion, midbrain, and striatum^65^. Induction of AD resulted in a significant loss of SIRT-1 neuroprotection, and poor staining was observed in different brain areas. DON and all V_A_ doses significantly increased SIRT-1 immunostaining, and VAMD/VAHD nearly restored SIRT-1 expression with results comparable to normal. In accordance, DON demonstrated SIRT-1 activation in some studies,it ameliorates induced brain microvascular endothelial cell dysfunction via the SIRT-1/FOXO3a/NF-κB pathways^66^. In addition, DON repressed high glucose-accelerated senescence in human umbilical vein endothelial cells through SIRT-1 activation^67^. SIRT-1 activation by V_A_ was also formerly verified in conditions other than AD^68,69^. V_A_ regressed ethanol-induced neuroinflammation by regulating NFκB and SIRT-1 expression^69^. Additionally, RA protected against high-fat diet-induced steatosis in mice by improving the antioxidant capacity through SIRT-1 activation^68^.

While the current findings demonstrate transcriptional upregulation of NGN2 and SOX-11, further studies are required to clarify the underlying molecular mechanisms. In particular, approaches such as promoter activity assays and chromatin immunoprecipitation (ChIP) analyses may help elucidate transcription factor binding dynamics and epigenetic regulation of their expression in the context of VA -associated neurogenesis-related processes.

To further assess cholinergic neuron regeneration and restoration of cholinergic transmission, the effect on brain levels of ACh, choline, choline acetyltransferase, and AchE was investigated. Rats with SCO-induced AD showed a significant decrease in the brain levels of ACh, choline, and choline acetyltransferase and an increase in AchE, reflecting decreased ACh synthesis and increased degradation. The significant cholinergic pathways alteration, accompanying the chronic non-selective muscarinic receptors blockade using SCO, is in line with the cholinergic hypothesis of AD. All treatments significantly enhanced the altered cholinergic pathways. The ability of DON to increase levels of ACh, its precursor (choline), and decrease its degradation is mostly a result of its primary mechanism of action, being an AchEI, rather than an indication of cholinergic neuron regeneration. The influence of V_A_, especially the medium dose, on cholinergic transmission was widely discussed in the literature. RA, as a potent cell differentiator, has been reported to facilitate the maturation of the ACh phenotype and to have robust ACh transmission enhancement in brain cholinergic neurons^70^. The addition of RA to rat sympathetic neurons in a cell culture medium significantly increased the specific activity of choline acetyltransferase^71^. Knocking out retinoid receptors in mice altered the electrophysiological processes crucial for learning and memory. Moreover, animals with induced V_A_ deficiency showed spatial memory task impairment and decreased hippocampal ACh release^72^. Further, retinoid receptors’ agonists enhanced cholinergic neurotransmission by increasing the expression of the vesicular ACh transporter gene and choline acetyltransferase gene^57^. Furthermore, V_A_ and RA increased the survival of cholinergic but not GABAergic neurons and increased the level of choline acetyltransferase^39^. The increased ACh synthesis and decreased degradation may point to the efficient cholinergic neurons’ regeneration and an increased number of functional cholinergic receptors requiring activation by their ligands, ACh and choline.

Histopathological and morphometric analyses were performed to assess the percentage of cortical neuronal degeneration. Cortical tissue was selected as AD is primarily characterized by impairment of cortical cholinergic innervation^73–75^.

All treatments reduced neuronal degeneration, with the lowest degeneration percentage observed in the VAMD-treated group, consistent with the previously reported findings. In parallel, VAMD significantly decreased hippocampal Aβ deposition and serum tau levels. Previous studies have reported that retinoids may reduce Aβ accumulation, potentially through modulation of retinoid signaling pathways and cholinergic function^39,76^. In addition, SIRT-1, a key regulator of cellular ageing, has been shown to mitigate Aβ-induced cellular^77^. Previous studies suggest that V_A_ may contribute to the clearance of tau aggregates, potentially through upregulation of heat shock protein 90, which is involved in facilitating tau degradation^78^. In addition, retinoids have been reported to modulate neuronal survival pathways, including regulation of steroidogenic acute regulatory protein expression, which may reduce amyloid precursor protein– and tau-related cytotoxicity^79^.Therefore, one possible mechanism underlying the observed reduction in Aβ deposition in the present study may involve SIRT-1–associated pathways. Similarly, the reduction in tau levels may be linked to retinoid-mediated regulation of protein homeostasis and SIRT-1 activity, both of which have been implicated in modulating tau expression and aggregation^80,81^. However, the precise molecular mechanisms require further investigation.

An additional aim of the present study was to explore the association between AD and liver fibrosis in the context of altered V_A_ homeostasis, as reflected by hepatic RBP expression. Accordingly, liver fibrosis was assessed histopathologically and biochemically. AD was associated with marked fibrotic changes, including increased fibrous tissue deposition, steatosis, hepatocyte ballooning, and elevated levels of TGF-β and hydroxyproline. The link between liver dysfunction and AD has been increasingly recognized. Liver fibrosis has been proposed as a potential risk factor for AD, with evidence showing increased Aβ and tau deposition in patients with non-alcoholic fatty liver disease^82^. Impaired hepatic clearance of Aβ and altered liver function may contribute to AD pathophysiology^75^. In addition, liver function parameters and fibrosis-related changes have been associated with circulating AD biomarkers^83^, Zhang^84^^,^ supporting the concept of a liver–brain axis in AD^85^.

In the present study, the observed hepatic changes may be associated with disrupted V_A_ homeostasis. Previous reports indicate that depletion of hepatic retinoid stores precedes HSC activation and fibrosis, while also impairing hepatocyte survival and regeneration^86^. Consistently, reduced hepatic retinoid signaling has been linked to steatosis and liver pathology, whereas retinoid supplementation may reverse these effects^87,88^.

Both DON and VA treatments improved liver fibrosis parameters. DON has been reported to exert variable hepatic effects, with hepatotoxicity mainly associated with long-term use^89^. Retinoids, on the other hand, have demonstrated hepatoprotective effects in multiple experimental models^86–88^. Although the highest hepatic RBP levels were observed in the VAHD group, the most pronounced improvement in fibrosis was achieved with VAMD. This finding may reflect a dose-dependent balance between therapeutic efficacy and potential toxicity. Previous studies support this observation, showing that lower V_A_ doses can improve hepatic structure and function, whereas higher doses may induce HSC activation and fibrosis^90,91^. One potential mechanism underlying these effects may involve SIRT-1, which has been reported to regulate HSC activation and fibrosis progression^92,93^. However, the precise mechanisms linking V_A_ signaling to hepatic outcomes in AD require further investigation.

In conclusion, SCO-induced AD was associated with reduced V_A_ levels in brain and liver tissues, accompanied by hepatic fibrosis. Administration of VAMD was associated with SIRT-1 overexpression, enhanced cholinergic signaling, and increased neurogenesis-associated activity, potentially mediated through upregulation of TFs involved in neuronal differentiation, including SOX-11 and NGN2. Additionally, VAMD reduced hippocampal Aβ deposition and serum tau levels and attenuated AD-associated fibrosis. These findings support a potential role for VAMD in modulating AD-related pathological processes.

Despite the promising findings, several limitations should be acknowledged. First, the evidence for neurogenesis-associated effects is based on indirect molecular markers (e.g., DCX, NGN2, and SOX-11) rather than direct lineage-tracing approaches; therefore, the cellular origin of the observed effects cannot be definitively determined. Second, the use of α-SMA as a marker reflects reactive cells rather than specific astrocyte identification, which limits precise characterization of the involved cell populations. In addition, the SCO-induced model reproduces key cholinergic deficits but does not fully represent the complex pathology of human AD. The precise molecular mechanisms linking V_A_ to SIRT-1 activation and TF upregulation were not fully elucidated and require further investigation. Moreover, long-term safety and functional behavioral outcomes were beyond the scope of this study. Future studies incorporating cell-type-specific co-localization, lineage-tracing techniques, and extended functional assessments are needed to validate and extend these findings.

## Data Availability

All data generated or analyzed during this study are included in this published article and its supplementary information files. RNA sequences analyzed during the current study are available in the GenBank repository, https://www.ncbi.nlm.nih.gov/genbank/ , accession numbers are included in Table 1.

## Abbreviations

Aβ: Amyloid-β
ACh: Acetylcholine
AChEIs: Acetylcholine esterase inhibitors
AD: Alzheimer’s disease
ALDH1A1: Aldehyde dehydrogenase 1 family member A1
α-SMA: Alfa-smooth muscle actin
DON: Donepezil
iPSCs: Induced pluripotent stem cells
DCX: Neurogenesis marker doublecortin
HDACs: Histone deacetylases
HSCs: Hepatic stellate cells
NGN2: Neurogenin 2
PD: Parkinson’s disease
qRT-PCR: Quantitative real time-PCR
RA: Retinoic acid
RBP: Retinol-binding protein
SCO: Scopolamine
SIRT-1: Sirtuin 1
TF: Transcription factors
TGF-ꞵ: Transforming growth factor-beta
V_A_: Vitamin A
VALD: V_A_ low dose
VAMD: V_A_ medium dose
VAHD: V_A_ high dose
